# Digital Cyanosis as a Manifestation of Upper Limb Atherosclerosis: A Case Report

**DOI:** 10.7759/cureus.76947

**Published:** 2025-01-05

**Authors:** Carolina Aispuro-Barrantes, Claudia M Garcia-González, Diego A Salinas-Orozco

**Affiliations:** 1 Internal Medicine, Hospital Universitario Dr. José Eleuterio González, Monterrey, MEX

**Keywords:** atherosclerosis, ballon angioplasty, cardio vascular disease, endo vascular intervention, peripheral arterial diseases, peripheral cyanosis, upper limp

## Abstract

In this case report, we present an atypical case of atherosclerosis manifesting as subclavian artery atherosclerosis in a 57-year-old male patient with a history of chronic smoking and dyslipidemia. The patient developed progressive cyanosis and vascular compromise in the fingers, characteristic symptoms of subclavian artery obstruction. The diagnosis was confirmed through Doppler ultrasound and computed tomography angiography, which revealed a 72% stenosis of the left subclavian artery. Balloon angioplasty was performed successfully and without complications, restoring blood flow and improving perfusion to the affected limb.

## Introduction

Cardiovascular diseases, including atherosclerosis, are a group of disorders that affect the heart and blood vessels, constituting the leading cause of death worldwide. According to the World Health Organization, an estimated 17.8 million people died from cardiovascular diseases in 2019, representing 32% of global deaths [1].

Atherosclerosis is a chronic and progressive disease characterized by the hardening and narrowing of the arteries due to the accumulation of lipids, inflammatory cells, and fibrous tissue in the arterial walls. This process begins with endothelial dysfunction that allows the infiltration of low-density lipoproteins (LDL) into the intimate layer of the arteries, where they oxidize and trigger a local inflammatory response. This reaction implies the activation of macrophages and the formation of foamy cells, which mark the first signs of so-called “fatty stretch marks” in the arteries [2]. Risk factors associated with this disease can be divided into traditional and non-traditional risk factors. Traditional risk factors are further classified into modifiable factors, including smoking, diabetes, hypertension, and dyslipidemia, and non-modifiable factors, such as age, sex, and family history. Non-traditional risk factors include obesity, sedentary lifestyle, sleep disorders, a stressful lifestyle, alcohol consumption, diet, apolipoproteins, autoimmune diseases, environmental pollution, socioeconomic status, pregnancy-induced hypertension/diabetes, and menopause. There are tools available to calculate an individual's cardiovascular risk based on their risk factors, such as Systematic Coronary Risk Evaluation 2 (SCORE2), the atherosclerotic cardiovascular disease (ASCVD) risk algorithm, Framingham, among others. These tools help assess the vascular risk of our patients and guide their treatment and follow-up when necessary [3,4].

This case report describes an atypical presentation of a common disease, atherosclerosis, in which only 2% to 9% of patients with atherosclerosis experience occlusion or stenosis of the upper extremity arteries [4]. We evaluated the clinical approach, diagnosis, treatment, and monitoring of the patient.

## Case presentation

**Figure 1:**
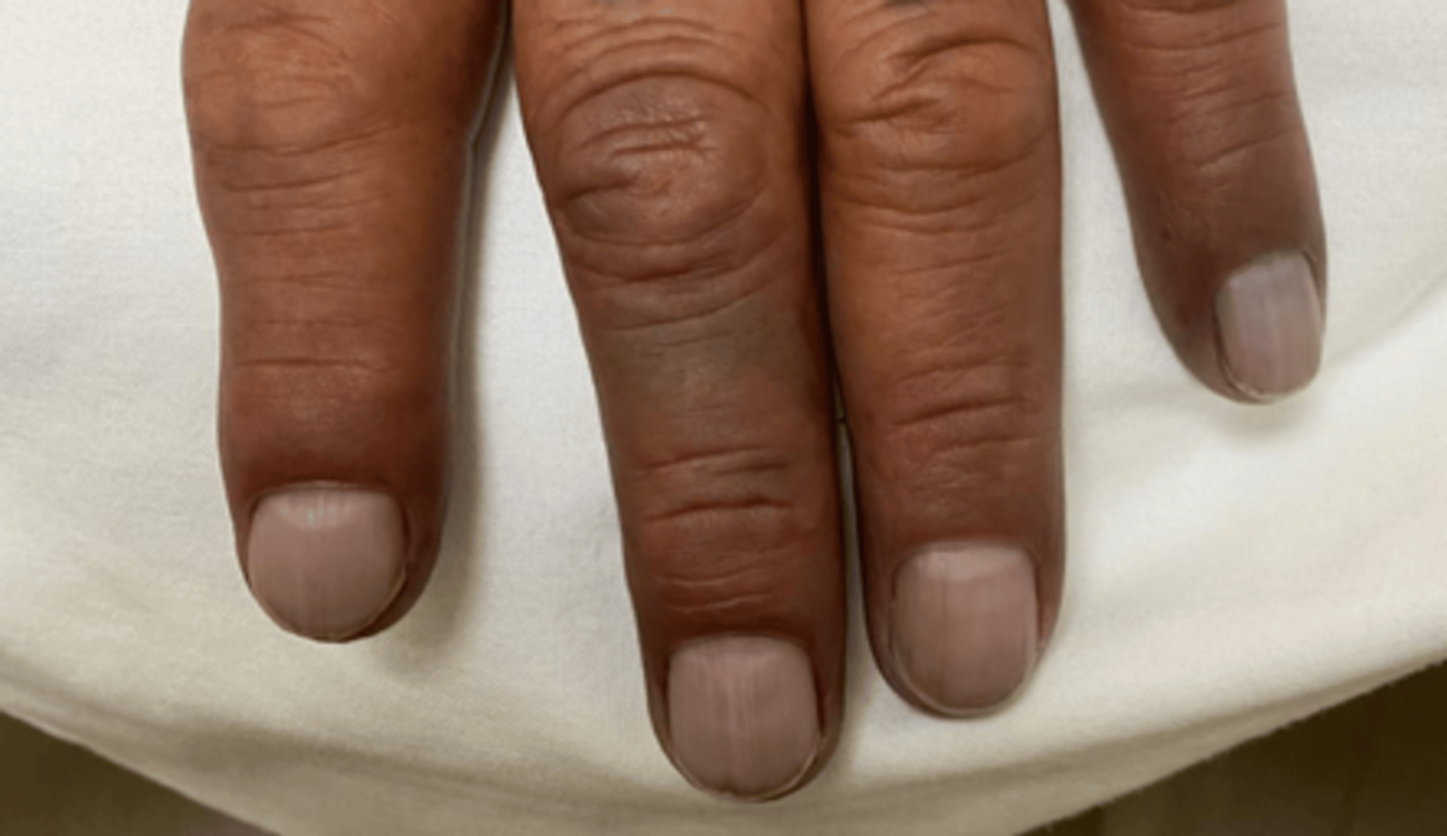
Back of left hand with cyanosis in second, third, fourth, and fifth fingers.

**Figure 2:**
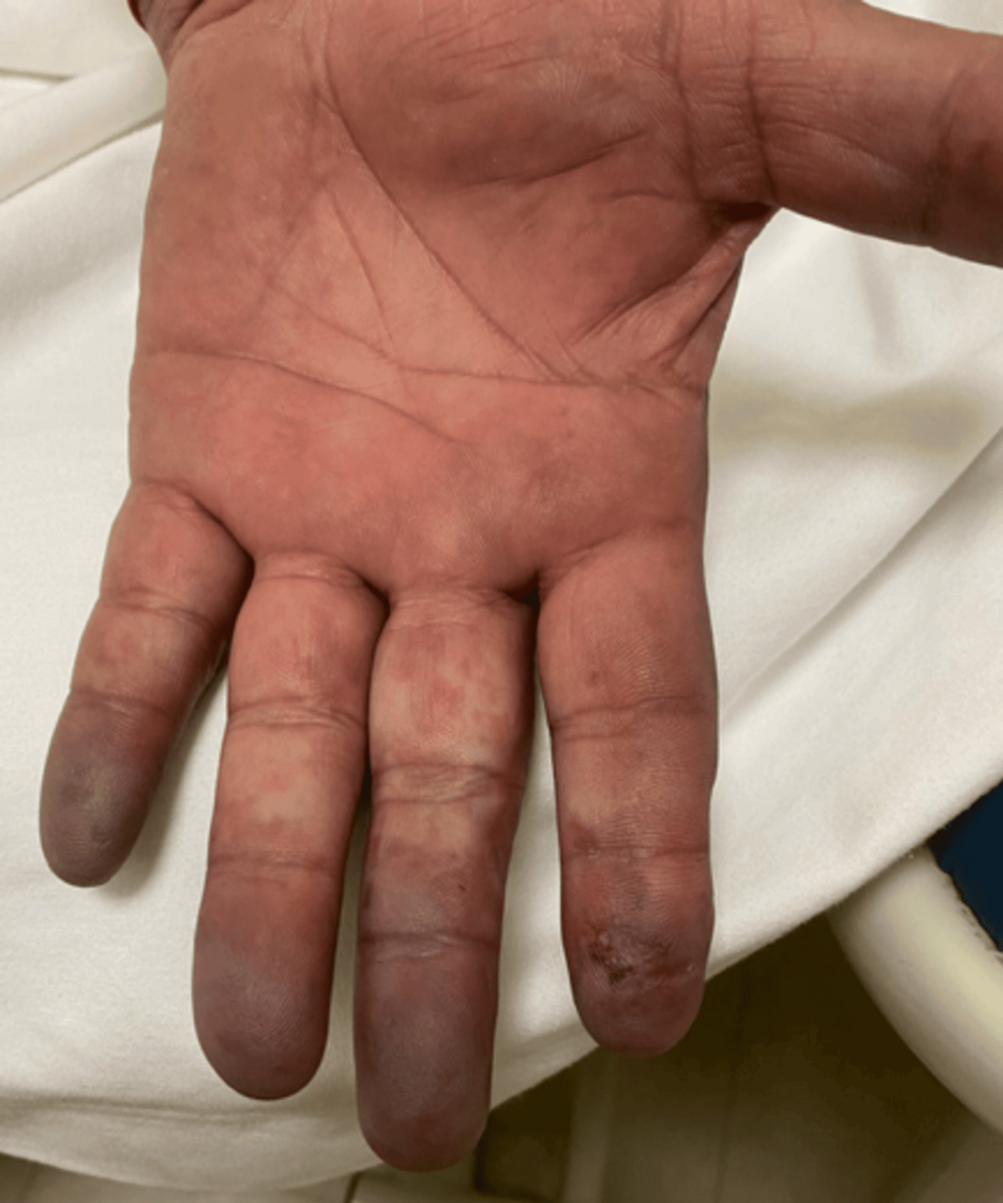
Palm of the left hand with pallor at the base of the third and fourth phalanx. Cyanosis in the distal phalanges and laceration of the pulp of the second finger can be observed.

We present the case of a 57-year-old male patient with a history of active smoking since age 12 at a rate of 15 packages/year, as well as active alcoholism from age 12 with a consumption of 63 g/week. The patient had a history of right supracondylar amputation secondary to osteomyelitis caused by trauma five years prior. Two months prior to admission, the patient experienced paresthesia in the pulp of the second finger of the left hand, which progressed to the third, fourth, and fifth ipsilateral fingers over approximately five days. One week after symptom onset, the affected fingers developed a violet coloration, and he continued with this symptomatology for two weeks. This symptom persisted for two weeks until, on the day of admission, the patient presented with spontaneous laceration of the second finger pulp (Figures 1, 2).

**Figure 3:**
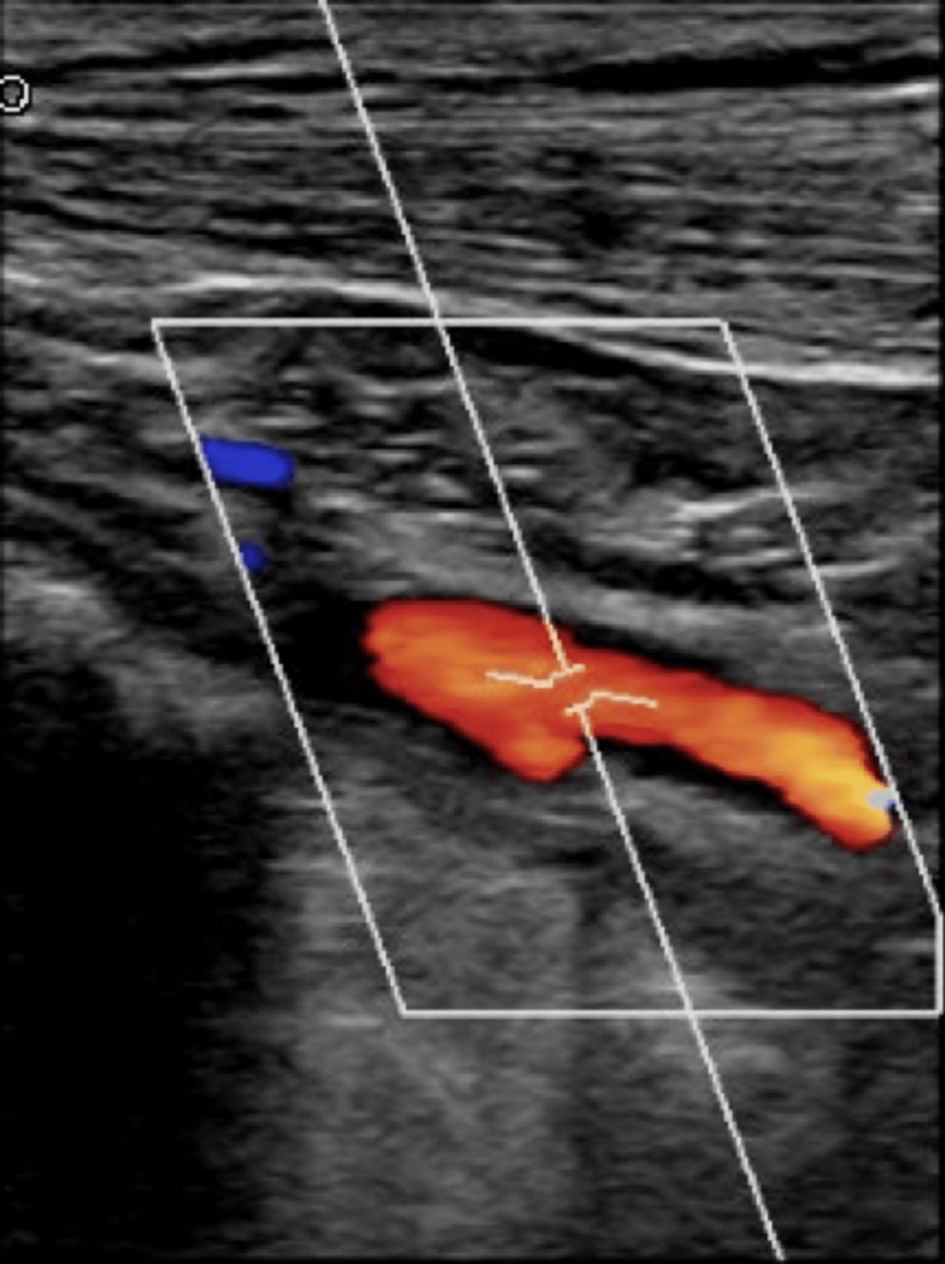
Subclavian artery with adequate flow on color Doppler assessment.

**Figure 4:**
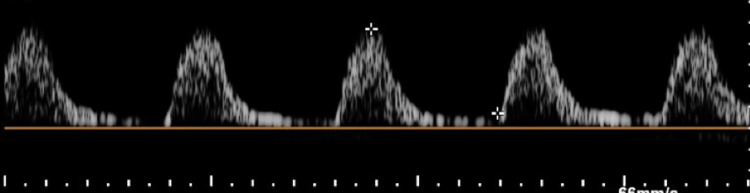
Monophasic pattern of intermediate resistance throughout the interrogated path of the left upper limb.

The patient was admitted to Hospital Universitario Dr. José Eleuterio González on April 9, 2024. Upon admission, vital signs were normal. However, based on initial symptoms, Raynaud's syndrome was suspected. It is important to note that the patient’s clinical characteristics were not consistent with Raynaud's syndrome, as the cyanosis was unilateral and the patient had not experienced the three characteristic phases of the syndrome. Therefore, the patient was reevaluated for signs suggestive of vascular occlusion in the affected extremity. Physical examination revealed a diminished perception of the left brachial and radial pulses, as well as discordant blood pressure (BP) between the extremities. The right upper extremity had a BP of 120/70 mmHg, while the left upper extremity showed a BP of 110/60 mmHg. Consequently, a Doppler ultrasound was performed on the affected extremity (Figures 3, 4), which found a monophasic pattern of intermediate resistance throughout the journey of the subclavian artery to the distal third of the brachial artery. 

**Figure 5:**
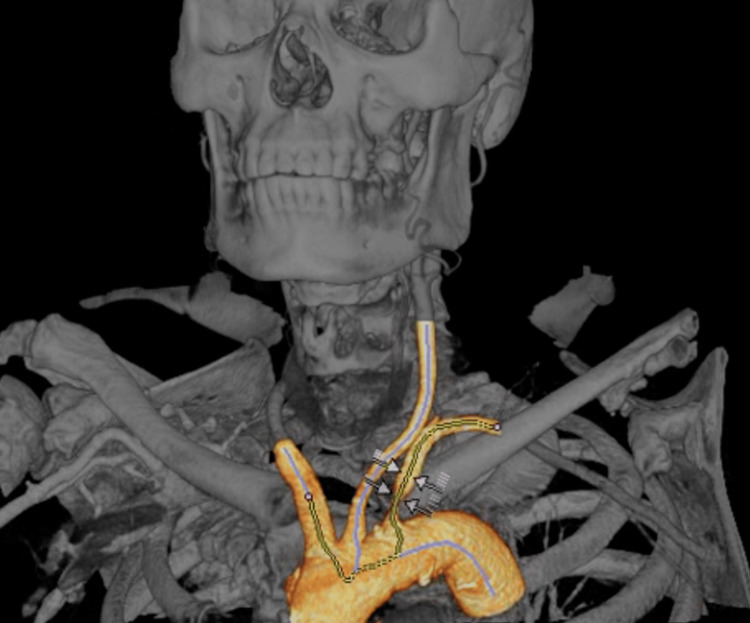
Three-dimensional reconstruction. Arrows indicate a circumferential stenosis caused by a mixed atheromatous plaque at the origin of the left subclavian artery, resulting in an approximately 72% reduction in lumen diameter.

**Figure 6:**
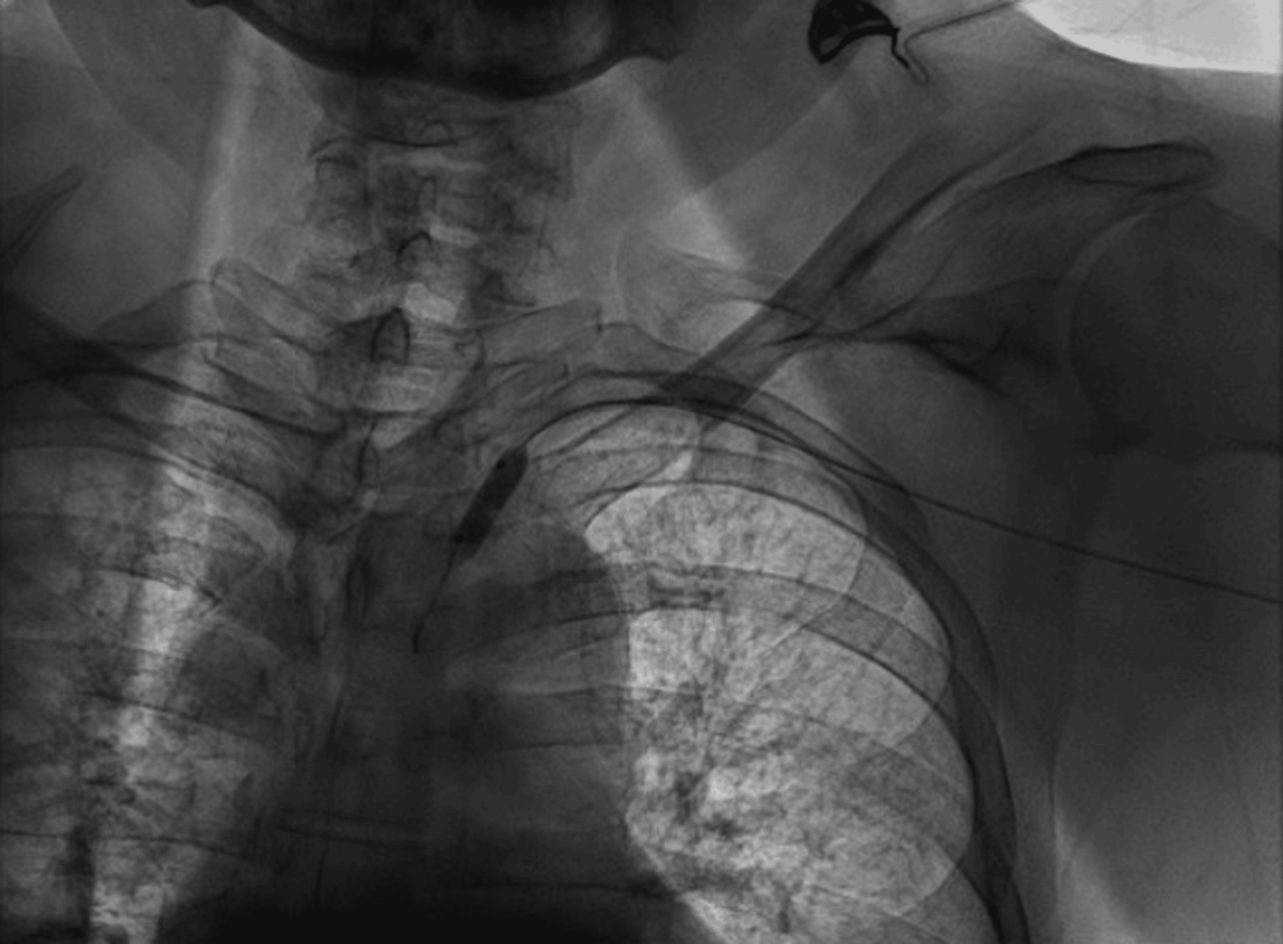
Balloon revascularization fluoroscopy.

**Figure 7:**
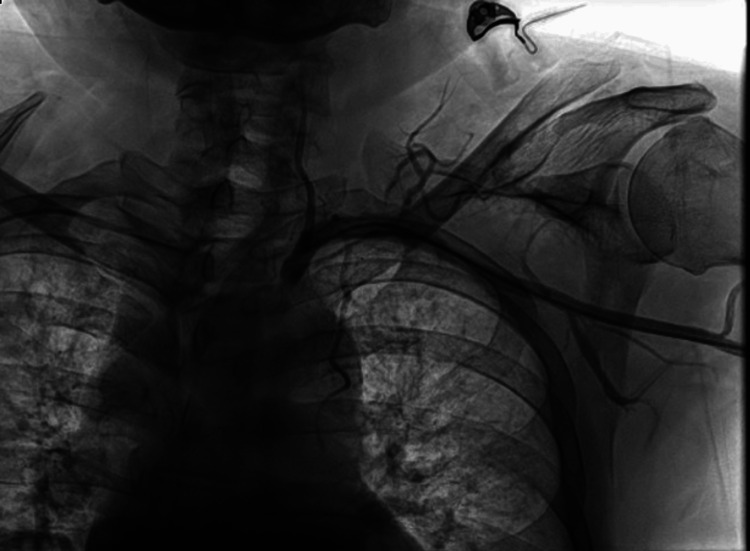
Control arteriography. An adequate passage of contrast medium can be observed, along with effective dilation of the stenotic area.

Laboratory testing revealed elevated cholesterol and triglycerides, prompting lipid profile testing (Tables 1-3). Computed tomography angiography (CTA) of the supra-aortic vessels was performed (Figures 5, 6), which revealed both atheromatous and non-atheromatous plaques at the origin of the left subclavian artery with 65% stenosis. Balloon angioplasty was performed, as it was the available endovascular treatment in our center (Figure 7). During his hospital stay, the patient received 80 mg atorvastatin orally every 24 hours and acetylsalicylic acid 100 mg orally every 24 hours. The patient was discharged 24 hours after the procedure, following medical monitoring. A 10-year cardiovascular atherosclerotic disease risk of 14.8% was calculated using the ASCVD risk estimator tool, so the patient was discharged with a treatment plan that included atorvastatin 80 mg orally every 24 hours. Smoking cessation was recommended, and follow a low-fat diet with an increased intake of fruits and vegetables, as suggested by our clinical nutrition team. A follow-up appointment was scheduled for six months, which will include a new CTA of the supra-aortic vessels and additional tests, including a complete blood count, blood chemistry, and lipid profile.

**Table 1:** Hematic biometry.

Test Parameter	Result	Units	Reference Value
Red Blood Cell	6.17	M/uL	4.04–6.13
Hemoglobin	19	g/dL	12.20–18.10
Hematocrit	57.4	%	37.7–53.7
Mean Corpuscular Volume	93	fL	80–97
Mean Corpuscular Hemoglobin	30.8	pg	27.0–31.2
Mean Corpuscular Hemoglobin Concentration	33.1	g/dL	29.9–34.2
Red Blood Cell Distribution Width	12.2	%	11.6–14.8
White Blood Count	11.3	K/uL	4.00–11.00
Neutrophils	7.26	K/uL	2.00–6.90
Lymphocytes	3.25	K/uL	0.60–3.40
Monocytes	0.665	K/uL	0.000–0.900
Eosinophils	0.081	K/uL	0.000–0.700
Basophils	0.082	K/uL	0.000–0.200
Platelets	195	K/uL	142.00–424.00
Medium Platelet Volume	7.9	fL	7.4–10.4

**Table 2:** Clinical chemistry test.

Test Parameter	Result	Units	Reference Value
Albumin	4.2	g/dL	3.2–5.5
Blood Uric Acid	8.2	g/dL	4.8–8.7
Total Bilirubin	0.7	mg/dL	0.2–1.0
Direct Bilirubin	0.1	mg/dL	0.0–0.2
Indirect Bilirubin	0.6	mg/dL	0.2–0.8
Cholesterol	222	mg/dL	130–200
Blood Creatinine	0.6	mg/dL	0.6–1.4
Phosphorus	3	mg/dL	2.5–4.6
Blood Glucose	61	mg/dL	60–100
Blood Urea Nitrogen	13	mg/dL	0.7[A2] –20
Serum Calcium	9.5	mg/dL	8.4–10.2
Triglycerides	267	mg/dL	35–150
Potassium	4.4	mg/dL	3.6–5.0
Sodium	139.3	mmol/L	135.0–145.0
Chlorine	102	mmol/L	101.0–110.0
Amylase	71	mOsm/L	28–100
Alkaline Phosphatase	90	U/L	38–126
Alanine Aminotransferase	52	UI/L	10–42
Aspartate Aminotransferase	26	UI/L	10–42
Lactic Dehydrogenase	105	UI/L	91–180

**Table 3:** Lipid profile. VLDL: very-low-density lipoprotein; HDL: high-density lipoprotein; LDL: low-density lipoprotein

Test Parameter	Result	Units	Reference Value
VLDL	54.2	mg/dL	2.0–40.0
HDL Cholesterol	35	mg/dL	29–71
Cholesterol	187	mg/dL	130–200
LDL Cholesterol	97.8	mg/dL	0.0–130.0
Triglycerides	271	mg/dL	35–150

## Discussion

Atherosclerosis begins with endothelial dysfunction, which initiates a series of pathological processes, including the retention of low-density lipoprotein (LDL) on the arterial wall, its oxidative modification, and the activation of inflammatory cells such as monocytes. These events culminate in the formation of foam cells and the accumulation of atheromatous plaques. These plaques, comprising lipids, fibrotic elements, and calcium deposits, cause the narrowing of blood vessels, obstruct blood flow, and predispose individuals to complications such as heart attack and stroke [2]. 

Key risk factors associated with this condition should be specifically assessed, including the history of diabetes mellitus, hypertension, dyslipidemia, or smoking. If any of these are present, physicians need to inquire about their onset, duration, and treatment. In the case of smoking, it is essential to ask about the number of cigarettes consumed per day and the total number of years of smoking [4,5]. Our patient had a history of chronic smoking, alcohol consumption, and dyslipidemia, which are significant risk factors for their current condition. Smoking has a profound impact on the development and progression of atherosclerosis through various mechanisms, including endothelial damage caused by toxic substances such as nicotine and carbon monoxide, induction of a chronic inflammatory response, alteration of lipid profiles by decreasing high-density lipoprotein (HDL) levels while increasing LDL and triglycerides, and promotion of platelet activation and aggregation [3]. In our patient, the laboratory tests revealed elevated levels of very-low-density lipoprotein (VLDL) cholesterol and triglycerides, which are of significant importance in this case, as VLDL cholesterol is synthesized in the liver and acts as a precursor to LDL cholesterol, the molecules identified as a significant risk factor in atherosclerotic disease. Since LDL can infiltrate the arterial wall, undergo oxidation, and promote inflammatory responses that result in the formation of atherosclerotic plaques, it is also important to highlight that our patient presented with hypertriglyceridemia upon admission. This has clinical significance, as elevated triglyceride levels are associated with an increased risk of atherosclerosis. This is due to their role in the exchange of esterified cholesterol from mature HDL and LDL with triglycerides from VLDL via the CETP enzyme, resulting in smaller, denser LDL particles, which are known for their high atherogenicity. The elevated alanine aminotransferase (ALT) levels observed are also significant in relation to the current condition, as they are associated with chronic inflammation that contributes to the development of atherosclerosis by promoting endothelial dysfunction through excessive production of reactive oxygen species, which can accelerate endothelial damage and facilitate the formation of atherosclerotic plaques. Elevated ALT levels are often associated with non-alcoholic fatty liver disease (NAFLD), which is considered a pro-atherogenic state due to its association with obesity, insulin resistance, and dyslipidemia. Although ALT is not a specific marker of atherosclerosis, elevated levels can signal underlying metabolic and hepatic dysfunctions that increase cardiovascular risk [6].

In 2018, the American College of Cardiology (ACC), in collaboration with the American Heart Association (AHA) and the National Heart, Lung, and Blood Institute, released updated guidelines for managing blood cholesterol to reduce the risk of atherosclerotic cardiovascular disease (ASCVD) in adults. These guidelines recommend assessing cardiovascular risk using the ASCVD risk calculator (ACC/AHA pooled cohort equations). This tool, developed based on data from five U.S. cohorts followed for a decade, estimates the 10-year risk of heart disease or stroke [5]. The SCORE2 tool is also recommended; however, this tool considers the country of origin in its variables, and Mexico is not included in its population. Therefore, the ASCVD risk estimator tool was used to calculate the patient’s cardiovascular risk. This tool categorizes risk into low (<5%), borderline (5-7.5%), intermediate (7.5-20%), and high (>20%), allowing for the selection of the most appropriate treatment for the patient based on these guidelines. In the case presentation, it was mentioned that during the patient’s hospital stay, atorvastatin 80 mg orally every 24 hours and acetylsalicylic acid 100 mg orally every 24 hours were administered. Given the patient’s intermediate risk, the use of 80 mg of atorvastatin orally every 24 hours is indicated. Other therapeutic options, such as rosuvastatin, are available; however, atorvastatin is the statin available in our center. The administration of acetylsalicylic acid was guided by the 2024 ESC guidelines, which indicate its use in cases of asymptomatic stenosis or in patients who cannot undergo a therapeutic procedure. In this patient’s case, the medication was administered while awaiting balloon angioplasty [4].

The arterial disease of the upper limbs due to atherosclerosis is primarily located at the level of the brachiocephalic trunk, as well as the subclavian and axillary arteries [7], as observed in our patient, who suffered occlusion of the left subclavian artery. The prevalence of this manifestation of atherosclerosis is estimated at 2% in the general population; however, it increases to 9% in cases of concomitant atheromatous disease of the lower extremities [3].

Clinical evaluation and physical examination are the first steps in diagnosing patients with upper limb atherosclerosis. Findings may include the absence or reduction of carotid, axillary, brachial, and radial pulses; carotid or subclavian bruits; and a systolic blood pressure difference of 10-15 mmHg between arms, where a difference greater than 15 mmHg increases the risk of cardiovascular death by 50%. Therefore, it is recommended to measure blood pressure in both arms [3].

Doppler ultrasound evaluation is the first step in screening and diagnosing these cases. Evaluation of the subclavian arteries can detect high-velocity flows indicative of >50% stenosis. Due to the proximal location of subclavian lesions, differentiating high-grade ostial stenosis from complete occlusion can sometimes be challenging. Single-phase post-stenotic flow and altered flow in the ipsilateral vertebral artery are common in cases of >70% proximal subclavian stenosis. Although Doppler ultrasound has a sensitivity of 88% and specificity of 95% for detecting stenosis >50%, these findings are not always conclusive. In such cases, CTA can be performed, which is an excellent option for visualizing lesions in supra-aortic vessels [3,8]. In our patient, Doppler ultrasound revealed monophasic flow, indicating medial stenosis, leading to a decision to perform a CTA. The CTA confirmed 72% stenosis.

Revascularization is indicated in symptomatic patients with transient ischemic attack/stroke, coronary subclavian steal syndrome, access dysfunction for ipsilateral hemodialysis, or deterioration in quality of life [5]. Revascularization in asymptomatic patients with planned coronary artery bypass grafting should be considered using the internal mammary artery, as well as in those with access for ipsilateral hemodialysis and in asymptomatic patients with significant bilateral subclavian stenosis or significant bilateral occlusion for adequate surveillance of blood pressure. Both endovascular and surgical procedures are available for revascularization [7-9]. Percutaneous angioplasty for subclavian artery stenosis is commonly performed with the placement of a stent. However, there is no conclusive evidence to determine whether stent placement is more effective than balloon angioplasty [7,8]. In patients with symptoms and indications for surgery or an endovascular procedure, prostanoid infusion or thoracic sympathectomy can be considered. In our patient, balloon angioplasty was chosen as the treatment since it is the endovascular procedure available at our center. According to the guidelines, follow-up should be conducted at least once a year, as the artery may re-stenose after balloon angioplasty even without an atherosclerotic plaque.

A Mediterranean diet, aerobic exercise, and lifestyle changes, such as smoking and alcohol cessation, are also recommended [4]. The patient and their family were counseled by our hospital’s clinical nutrition team, who provided a list of foods the patient could consume within their economic means, along with meal preparation suggestions. Exercise counseling was not provided because the patient has a supracondylar amputation without a prosthesis, which makes physical activity unfeasible.

## Conclusions

This case illustrates a typical presentation of subclavian atherosclerosis in a patient with significant risk factors, including chronic smoking and dyslipidemia, and highlights the importance of early detection and treatment in cases of atherosclerosis with unusual symptoms. Progressive cyanosis and vascular commitment in the hand reflect the seriousness of arterial obstruction and emphasize the importance of an early diagnosis and adequate management to prevent major complications. Balloon angioplasty turned out to be an effective intervention in this case, restoring blood flow and improving perfusion in the affected limb. In addition, the case underlines the need for lifestyle modification and long-term pharmacological treatment strategies to reduce cardiovascular risk, such as statins with antiplatelets, along with the suspension of smoking and a healthy diet. These approaches are essential to avoid the progression of atherosclerosis, reduce cardiovascular risk, and improve the patient’s quality of life.
